# The association between ethnicity, environmental and lifestyle factors and chronic disease in the development of pseudoexfoliation syndrome

**DOI:** 10.12669/pjms.37.2.2216

**Published:** 2021

**Authors:** Saba Ali Arif, Muhammad Ifraheem Khan, Fatimah Nauman, Mohammad Ali Arif

**Affiliations:** 1 Saba Ali Arif, MBBS. Pakistan Institute of Medical Sciences (PIMS), Pakistan Institute of Medical Sciences (PIMS), Islamabad, Pakistan; 2 Muhammad Ifraheem Khan, MBBS. Layton Rahmatullah Benevolent Trust, (LRBT), Karachi, Pakistan.; 3 Fatimah Nauman, MBBS. Mohammad Ali Arif, FCPS, MRCP.Pakistan Institute of Medical Sciences (PIMS), Islamabad, Pakistan; 4 Mohammad Ali Arif, FCPS, MRCP. Pakistan Institute of Medical Sciences (PIMS), Islamabad, Pakistan

**Keywords:** Pseudoexfoliation syndrome, Ethnic groups, Environment, Pakistan, lifestyle

## Abstract

**Objectives:**

To determine the association between ethnicity, environmental factors, lifestyle factors, chronic diseases and pseudoexfoliation syndrome (PEX).

**Methods:**

A case control study conducted at four major hospitals in Pakistan from January to November 2019, with 241 cases and 294 controls, aged ≥ 40 years, who were administered a questionnaire assessing demographics, lifestyle factors, chronic diseases and ethnicity. Multivariate binary logistic regression was applied to calculate the odds ratio between cases and controls.

**Results:**

PEX was found to be positively associated with ethnicity (p<0.001), time spent outdoors (p<0.001), educational status (p<0.001), asthma (p<0.001), mean age (p<0.001), daily tea intake (p=0.003), weighted maximum temperature (p<0.001) and weighted mean temperature (p=0.004). Poor association was found with weighted latitude (p=0.526) and weighted minimum temperature (p=0.079). Odds ratios for patients with asthma (OR=7.366, regression coefficient=1.993, p<0.001) Pathan ethnicity (OR=1.616, regression coefficient=0.48, p=0.016) and mean weighted temperature (OR=0.907, regression coefficient-0.097, p=0 .000) were significant in diagnosed cases of PEX.

**Conclusion:**

Individuals with Pathan ethnicity and asthmatics should be made aware of the risk of developing PEX and the importance of periodic screening. Limiting exposure to sunlight and cold and reducing the intake of tea may help in reducing in the chances of developing PEX.

## INTRODUCTION

Pseudoxfoliation Syndrome (PEX) is a multisystem disorder with progressive deposition of extracellular fibrillary material throughout the body. The most significant and well researched impact of the disorder is on the eye, with associations with a multitude of ocular pathologies, such as early cataract formation, secondary open angle glaucoma, iris sphincter atrophy and fibrosis, zonular fragility and reduced endothelial cell density.^1^ PEX also predisposes an individual to increased risk of complications from cataract surgery such as corneal decompensation, posterior capsular rupture, vitreous loss, zonular rupture, intra ocular pressure spikes, increased incidence and severity of post-operative inflammation, posterior capsular opacification, capsular contraction, intraocular lens (IOL) subluxation and late IOL dislocation.^1^

The severe visual impairment and blindness as a consequence of PEX are mainly related to cataract and glaucoma. PEX is the most common identifiable cause of glaucoma worldwide, with the prognosis being worse than primary open angle glaucoma.^1^ Glaucomatous visual deterioration often goes undetected until the disease is advanced, leading to serious visual morbidity. In a hospital based study conducted in Pakistan, 20% of patients with pseudo exfoliation glaucoma had bilateral blindness (visual acuity< 3/60 in the better eye) at the time of initial presentation.^2^ Early detection of the disorder is of paramount importance in order to institute early treatment to prevent visual loss.

The prevalence of PEX varies between countries and among various ethnic groups, from 0% in Eskimos to 21% in Finns over 60 years of age.^3^ In the Pakistani population the overall prevalence, irrespective of ethnicity, has been reported to be 1.81%.^4^ Various studies have been conducted in Pakistan on the ocular associations, management strategies, surgical complications and genetic polymorphisms related to PEX^5^-^7^ but none have been carried out on the ethnic, demographic, environmental and systemic disease associations of the disorder. International studies have shown the prevalence of PEX to demonstrate ethnic variability;^3^,^8^ and PEX has been found to be associated with environmental and demographic factors such as latitude and ambient temperatures;^9^-^11^ with social factors such as smoking and intake of tea;^12^ and with various systemic disorders such as hypertension, ischemic heart disease, asthma, and dementia.^13^

In our study we analyze the association between these factors and PEX in the Pakistani population. With identification of high risk ethnic groups, screening programmes can be focused on these populations for identification of cases; and awareness can be created regarding the importance of regular screening for early diagnosis in these groups. Identification of positive association of PEX with certain environmental and social factors can help reduce the disease burden by lifestyle modification. Moreover, in case of association with systemic disease, multidisciplinary approach can aid in identifying undiagnosed cases of PEX and individuals with PEX can be screened for associated disorders.

## METHODS

This case control study was conducted at four major hospitals in Pakistan, namely Pakistan Institute of Medical Sciences, Islamabad; and Layton Rahmatullah Benevolent Trust hospitals in Shahpur (Sargodha), Quetta and Karachi, from January to November 2018. The study was approved by the Ethical Review Boards of the hospitals (F1-1/2015/ERB/SZABMU/178 and LRBT/FBEH/ERC/2019 respectively). Sample size was calculated by using prevalence of PEX taken as 2%,^4^ Confidence Level 95% and absolute precision 5%, suggested sample size was 134 cases and 134 controls.

Five hundred and thirty-five participants (241 cases and 294 controls), aged 40 and above, who visited the mentioned hospitals were selected via consecutive, non-probability sampling. Cases were identified based on presence of fibrillary material along the pupillary margin or on the anterior capsule of the lens on slit lamp examination after pupillary dilation. Age matched participants without PEX were selected as controls. A self-constructed questionnaire was administered to all participants, enquiring about age, education, ethnicity (Punjabis, Sindhi, Balochis, Pathans and Kashmiris), prior and current place of residence, years spent at each residence, hours spent outdoors daily, presence of chronic diseases (diabetes mellitus, hypertension, ischemic heart disease, chronic kidney disease and asthma), tea consumption, and smoking. The questionnaires were administered in a uniform manner by trained interviewers, masked to the ocular status of the participants.

Climatic data for the areas of residence was obtained with special permission from the Pakistan Meteorological Department. Data on latitude of residence was obtained online via https://www.mapsofworld.com.^14^ Data was analyzed using Statistical Package for the Social Sciences (SPSS) version 25. Qualitative variables were compared using chi square test. Multi-collinearity between quantitative variables was tested through Pearson correlation and multivariate binary logistic regression with forward Wald criteria was used to calculate the odds ratio.

## RESULTS

A total of 535 participants (306 females and 229 males) were enrolled with 241 cases and 294 controls. No significant relationship was found between gender and PEX (p=0.084). One hundred thirty people had migrated to another city during their lifetime, with 66.2% having migrated to Islamabad from their original place of residence.

Out of the qualitative variables assessed, PEX was found to be significantly associated with ethnicity (p<0.001), time spent outdoors(p<0.001), educational status(p<0.001), asthma(p<0.001), and chronic disease(p<0.001) (Table-I).

**Table-I:** Descriptive analysis of qualitative variables.

*Variable*	*Categories*	*Pseudoexfoliation syndrome*		*Total participants*	*P-value*

		No	Yes		
Ethnicity	Others	226	148	374	0.000
	Pathan	68	93	161	
	<4 hours	184	93	277	
Time spent outdoors	4-8 hours	88	124	212	0.000
	>8 hours	22	24	46	
Education	Illiterate	158	180	338	0.000
	Literate	136	61	197	
Gender	Male	116	113	229	0.084
	Female	178	128	306	
Smoking	No	268	207	475	0.055
	Yes	26	34	60	
Diabetes	No	216	167	383	0.287
	Yes	78	74	152	
Hypertension	No	176	138	314	0.543
	Yes	118	103	221	
Ischemic Heart Disease	No	268	218	486	0.78
	Yes	26	23	49	
Asthma	No	290	217	507	0.000
	Yes	4	24	28	
Chronic kidney disease	No	292	239	531	0.842
	Yes	2	2	4	
Chronic diseases	No	142	76	218	0.000
	Yes	152	165	317	

Analysis of quantitative variables found mean age(p<0.001), number of cups of tea per day(p=0.003), weighted maximum temperature (p<0.001) and weighted mean temperature (p=0.004) to be significantly different between cases and controls. No significant difference was found between cases and controls in cases of weighted latitude and weighted minimum temperature (p-value 0.526 and 0.079 respectively) (Table-II).

**Table-II:** Comparative analysis of quantitative variables.

*Variables*	*Exfoliation syndrome*		*Overall*	*P-value*

	*Yes*	*No*		
Age	61.81±10.667	58.52±10.318	60.00±10.595	0.000
Tea (cups per day)	2.56±1.313	2.24±1.188	2.38±1.254	0.003
Weighted Latitude	32.88±2.441	32.767±1.617	32.819±2.029	0.526
Weighted Minimum Temperature	9.815±3.56	10.327±3.138	10.096±3.351	0.079
Weighted Maximum Temperature	31.379±3.209	32.45±3.671	31.968±3.508	0.000
Weighted Mean Temperature	21.382±3.531	22.256±3.659	21.862±3.531	0.004

Chi square test was applied to assess the independence between categorical variables, the detailed results of which are given in Table-III. Multicollinearity between quantitative variable was tested through Pearson correlation, with details given in Table-IV.

**Table-III:** Test of independence between ethnicity and other categorical variables.

*Variable*	*Categories*	*Ethnicity*		*P-value*

		*Others*	*Pathan*	
Time spent outdoors	<4 hours	214	63	0.000
	4-8 hours	135	77	
	>8 hours	25	21	
Education	Illiterate	220	118	0.001
	Literate	154	43	
Asthma	No	353	154	0.546
	Yes	21	7	
Chronic diseases	No	161	57	0.099
	Yes	213	104	

**Table-IV:** Test of multicollinearity between independent quantitative variables.

*Variables*	*Weighted maximum temperature*	*Weighted mean temperature*	*Age*
Tea (cups per day)	-0.142 (0.001)	-0.122 (0.005)	0.022 (0.617)
Weighted maximum temperature	1	0.95 (0.000)	0.019 (0.66)
Weighted mean temperature		1	0.023 (0.589)

Due to existence of multicollinearity between the significantly associated variables, all of the variables could not be included in the analysis. Multivariate binary logistic regression with forward Wald criteria was applied (Table-V). The results of Negelkerke R2 and Cox & Snell R2 with this model were 0.154 and 0.115 respectively, with true prediction of 63.7%. Intercept could not be used in our final model due to the issue of goodness of fit, tested through Hosmer and Lemeshow tests, which showed that our model is best fit with the value 0.749.

**Table-V:** Final model of multivariate binary logistic regression.

*Variable*	*Regression coefficient (β)*	*P-value*	*Odds ratio*	*95% Confidence interval for odds ratio*	

				*Lower*	*Upper*
Ethnicity	0.480	0.016	1.616	1.092	2.391
Asthma	1.993	0.000	7.336	2.443	22.029
Chronic disease	0.536	0.004	1.709	1.181	2.473
Age	0.023	0.001	1.023	1.010	1.037
Weighted mean Temperature	-0.097	0.000	0.907	0.874	0.942

Odds ratios for patients with asthma (OR = 7.366, regression coefficient 1.993, p<0.001) Pathan ethnicity (OR=1.616, regression coefficient 0.48, p=0.016) and mean weighted temperature (OR=0.907, regression coefficient -0.097, p = 0.000) were significant in PEX cases.

## DISCUSSION

Out of the five major ethnicities of Pakistan, we found the prevalence of PEX to be highest in the Pathan population, with 1.6 times increased odds of developing PEX, even after adjusting for the influence of climatic and geographic factors. Our findings were similar to the Ural Eye and Medical Study conducted in Russia that reported the prevalence of PEX to be greater in Russians as compared to non-Russians irrespective of place of habitat.^8^ The influence of ethnicity is also demonstrated by the prevalence of 0% PEX in Eskimos,^3^ despite their exposure to high amount of solar radiation and low temperatures, both of which are reported to be positively associated with PEX.

Our study found exposure to higher temperatures to have a negative association with the development of PEX. In the study by Stein et al, the hazard of PEX decreased by 9% for every 1° increase in July high temperature.^9^ The prevalence of PEX is also highest in countries with colder mean temperatures, with prevalence in Scandanavian countries of around 20%.^3^ An explanation is that PEX being a nucleation reaction, is prone to occur at colder temperatures. Owing to the lack of vascularity of the cornea, lens and aqueous humor, the anterior segment of the eye is susceptible to changes in environmental temperatures, resulting in the aggregation and precipitation of exfoliatory material in the eye.

We found greater amount of time spent outdoors during the day; predisposing one to increased exposure to solar radiation and extremes of temperature; to be positively associated with PEX. Pasquale et al reported that every hour spent outdoors, during the summer, between 10 am and 4pm, averaged over a lifetime, was associated with 4% increased odds of PEX.^10^ Arakaki et al also reported PEX to be positively associated with working outdoors.^11^ The association is because ultraviolet exposure upregulates the expression of the lysyl oxidase like 1(LOXL1) gene, genetic variants of which are associated with the development of PEX.^15^

Increasing latitude has been reported to be positively associated with development of PEX due to the lesser angle of incidence of light rays with respect to the horizontal, resulting in greater entry of UV rays in to the eye. However, latitude and PEX were not found to be directly related in our study. Mean weighted latitude was significantly higher in Pathans as compared to other ethnic groups (p<0.001), therefore ethnicity and latitude could not be used in a single model as a predictor. Kang et al found residence at age 15 years was more strongly implicated in the development of PEX as compared to other ages.^16^ We did not assess the influence of residence at a particular latitude in relation to age, which may have resulted in the null association between latitude and PEX. Moreover, the number of sunny days in an area, the individual’s sunshine exposure and altitude have also shown to modify the association between latitude and PEX^9^ which may have served as confounding variables in our study.

We found literacy to be associated with lower incidence of PEX (p<0.001). Vijaya et al also reported illiteracy to be a risk factor for the development of PEX.^17^ The explanation for this could be that the literate are involved in desk jobs as opposed to field work and farming, resulting in lesser solar exposure. Also the literate, due to awareness of the harmful effects of solar radiation, tend to use solar protection in the form of hats and sunglasses. Pasquale et al reported the use of sunglasses to be protective against the development of PEX.^10^

A strong positive association was found between self-reported asthma and PEX (p<0.001) in our study, with asthmatics having seven times greater odds of developing PEX than non-asthmatics. Batur et al, reported poorer pulmonary functions in individuals with PEX as compared to those without,^18^ while Taylor et al found an increased risk of chronic obstructive pulmonary disease (COPD) diagnosis in individuals with PEX.^19^ It was hypothesized that impaired elastogenesis associated with LOXL1 mutations in PEX may alter the repair processes in the lung, leading to pulmonary damage, especially after an insult such as cigarette smoking. Higher tea consumption was also found to be positively associated with PEX(p=0.003). Consumption of high doses of polyphenols present in tea are associated with elevated homocysteine levels in plasma and aqueous humor.^12^ Homocysteine augments the expression of elastin and causes structural changes promoting its aggregation, thereby contributing to development of PEX.^20^ It also causes vascular damage and oxidative stress, thereby contributing to the development of exfoliation glaucoma and other systemic disorders.^21^

### Strength of the study

The strength of our study was that it was the first of its kind studying the influence of ethnic variation in Pakistani population on the risk of development of PEX. Other factors assessed had also not been previously studied in the Pakistani population. Interviewer bias was ruled out by the use of trained interviewers masked to the ophthalmic status of the participants. Accurate climatic data was obtained with special permission from the Pakistan Meteorological Department.

### Limitations of the study

Limitations included recall bias by the participants regarding the number of hours spent outdoors. The participants may have had undiagnosed chronic diseases and the presence or absence of chronic diseases was documented based on self-reporting. Detection rate of PEX between the centers may have differed although all examiners were qualified ophthalmologists. Finally, the average climatic conditions of the locality may not have reflected each participant’s actual exposure.

## CONCLUSION

The Pathan population may be educated regarding increased risk of developing PEX and thereby undergoing regular ophthalmic screening to preempt possible future complications. Lifestyle modifications such as limiting exposure to sunlight and cold and reducing the intake of tea may help in reducing in the chances of developing PEX. Those with asthma and age above 40 may be advised ophthalmic evaluation for early detection of PEX. Likewise, subjects with PEX may be strongly advised to avoid cigarette smoking due to greater susceptibility to pulmonary damage.

### Authors’ Contributions:

**SAA:** Conception, design, drafting and editing.

**MIK, FN, MAA:** Design, data collection and revision.

**SAA** accountable for all aspects of the work in ensuring that questions related to the accuracy or integrity of any part of the work are appropriately investigated and resolved.
